# An update on the prevalence of eating disorders in the general population: a systematic review and meta-analysis

**DOI:** 10.1007/s40519-021-01162-z

**Published:** 2021-04-08

**Authors:** Jie Qian, Ying Wu, Fanxiao Liu, Yikang Zhu, Hua Jin, Hongmei Zhang, Yumei Wan, Chunbo Li, Dehua Yu

**Affiliations:** 1 Shanghai Yangpu District Central Hospital, Tongji University School of Medicine, Shanghai, China; 2 Shanghai Tianyou Hospital, Shanghai, China; 3 Shandong Provincial Hospital Affiliated to Shandong First Medical University, Jinan, China; 4 Shanghai Mental Health Center, Shanghai Jiao Tong University School of Medicine, Shanghai, China; 5 Shanghai Yangpu District Mental Health Center, Shanghai, China; 6 Shandong Mental Health Center, Jinan, China

**Keywords:** Eating disorders (EDs), Anorexia nervosa (AN), Bulimia nervosa (BN), Binge eating disorder (BED), Prevalence, General population

## Abstract

**Objective:**

To update the prevalence of eating disorders in the general population before 2021 and to analyze the distribution characteristics at different times and in different regions and sexes, as well as the diagnostic criteria.

**Methods:**

Based on the method from a previous report by the authors, studies were identified from the following databases: PubMed/Medline, PsycINFO, ISI Web of Knowledge, Ovid and the 4 most important Chinese databases. Articles in English and Chinese before 2021 were retrieved. The data retrieved at this time were pooled with the data from a previous report for analyses.

**Results:**

Thirty-three studies were identified, which included 18 studies supplemented in this retrieval. The pooled lifetime and 12-month prevalence of eating disorders were 0.91% (95% CI, 0.48–1.71) and 0.43% (95% CI, 0.18–0.78), respectively. The pooled lifetime and 12-month prevalence of the subgroup EDs (any), which covers all types of eating disorders, were 1.69% and 0.72%, respectively. The lifetime prevalence of AN, BN and BED was 0.16% (95% CI, 0.06–0.31), 0.63% (95% CI, 0.33–1.02) and 1.53% (95% CI, 1.00–2.17), respectively. The lifetime prevalence of EDs in Western countries was 1.89%, and was high at 2.58% in females. Prevalence studies using DSM-5 criteria were scarce.

**Conclusions:**

The prevalence of eating disorders might be underestimated thus far. Not all types of EDs were included in a majority of epidemiological surveys, and the prevalence rates of the new types of EDs were significantly higher. Eating disorders were especially common in Western countries and in females. New diagnostic criteria should be used to comprehensively assess all types of eating disorders.

**Level of evidence:**

1, systematic review and meta-analysis.

## Introduction

Eating disorders (EDs) are a group of syndromes characterized by eating behaviors and psychological disorders accompanied by weight changes and/or social disorders that have a significant influence on quality of life and social function [1, 2]. Moreover, individuals with eating disorders may develop severe somatic complications that can cause a higher risk of suicide [3] and increased mortality rates, especially anorexia nervosa (AN) [4], one of the main types, with a fatality rate as high as 5–20% [5].

The criteria of eating disorders evolved over time. In the Diagnostic and statistical Manual of Mental Disorders, 4th edition (DSM-IV) [6], eating disorders include anorexia nervosa (AN), bulimia nervosa (BN), and eating disorder not otherwise specified (EDNOS). EDNOS is a complex diagnosis recognized as all eating disorders that do not meet the diagnostic criteria of AN or BN or cannot be categorized into either of the two. Binge eating disorder (BED) is also classified into EDNOS and is listed in appendix. However, recent studies found that the impact of physical and psychological damage caused by EDNOS is no less than that caused by the classic eating disorder types AN or BN [7]. In the DSM-5 issued in 2013 [8], the diagnostic categories of eating disorders were expanded to “feeding and eating disorders”, in which feeding and eating disorders first seen in infants and early childhood were included. Binge eating disorder (BED) was listed individually in the diagnostic criteria. Exclusive of AN, BN and BED, the rest are classified as other specified feeding and eating disorder (OSFED) and unspecified feeding and eating disorder (UFED). OSFED refers to eating disorders that can lead to patients' clinical suffering or damage to social function, such as atypical AN, atypical BN, atypical BED, purging disorder, and night eating syndrome, which do not meet the criteria for AN, BN or BED. Other eating disorders that cause clinical suffering or impaired social function in patients but do not meet the diagnostic conditions listed above are classified as UFED. Apart from the changes in classification, the diagnostic criteria of all types of eating disorders were relaxed in DSM-5. The weight loss requirement has been relaxed, and the requirement of “amenorrhea” has been removed in AN. In the diagnosis of BN, the frequency of binge eating or unduly compensational behavior was lowered from twice a week to once a week. Similarly, the frequency of binge eating in BED diagnostic criteria has also been lowered to once a week (Table 1).

**Table 1:** Differences in the diagnostic criteria for eating disorders between DSM-IV and DSM-5

Types of EDs	Diagnostic items	DSM-IV	DSM-5
AN	Weight	Lower than 85% of normal weight/body mass index (BMI) ≤ 17.5 kg/m^2^	Lower than the lowest value of normal weight/ / lower than the lowest predictive value of children or juvenile
	Amenorrhea	Specified	N/A
	Course	Not mentioned	3 months
BN	Frequency of bulimia nervosa and compensation behavior	Twice a week	Once a week
BED	Diagnosis	Listed in the appendix	Listed formally in the diagnostic classification
	Frequency of binge eating disorder	Twice a week	Once a week
EDNOS	Diagnosis	Specified	N/A
OSFED	Diagnosis	N/A	Specified
UFED	Diagnosis	N/A	Specified

Eating disorders had been claimed to be represented predominantly in Western countries and women in the past, which may be due to cultural beliefs and attitudes in some aspect [9]. With industrialization and globalization, many regions reported increasing incidence rates of eating disorders [10, 11]. What’s more, the reported prevalence of eating disorders in the general population varied widely all along, which ranges from 0.1% [12] to 3.8% [13]. To address these issues, a systematic review on the prevalence of eating disorders in the general population was performed in 2013 by the author [14]. It showed that there were serious limitations in the available epidemiological data, especially from low- and middle-income countries, including China. Only 15 studies were included during the past 30 years since 2013. The pooled lifetime prevalence of eating disorders was 1.01%, but the specific types of eating disorders varied among the studies included. The prevalence of eating disorders was higher in women, and in Western than Asian countries, but South Korea was the only Asian country. The prevalence tended to increase over time but may also be caused by changes in the diagnostic criteria.

With the increasing attention to eating disorders in recent years, more and more related studies have emerged. Epidemiological studies have also increased, with many from the Asia countries and some even reporting high prevalence rates [15, 16]. More researches in men have also been reported, and the view that women are dominant seems to have wavered [4, 17]. In the meantime, some studies have indicated that the resulting prevalence by using DSM-5 is different from that by using DSM-IV [17–20]. In order to better understand the prevalence and the distribution characteristics of eating disorders, this study updated the systematic review on the prevalence of eating disorders in the general population. Thus, the main objectives of this study are (1) to update the prevalence of eating disorders and various types in the general population before 2021; (2) to analyze the distribution characteristics at different times and in different regions and sexes, as well as the diagnostic criteria between DSM-IV and DSM-5.

## Method

### Literature retrieval strategies

Previous research methods were followed in this study. The retrieved databases included PubMed/Medline, PsycINFO, ISI Web of Knowledge, Ovid and the Chinese Databases Chinese National Knowledge Infrastructure, Chongqing VIP database for Chinese Technical Periodicals, WANFANG DATA, and China BioMedical Literature Services System (SinoMed). The dates of retrieval were from 1 May 2013 to 31 January 2021. Only English and Chinese articles are included.

Other than the key words ‘eating disorders’, ‘anorexia nervosa’, ‘bulimia nervosa’, ‘prevalence’, and ‘epidemiology’ in both English and Chinese used in our previous study, we added a new key word ‘mental’ in Chinese in this study. Furthermore, articles before May 2013 were additionally retrieved by using the key words 'mental' and ‘prevalence/epidemiology’. All references in the included literature were then retrieved manually.

After all duplicated studies retrieved from the databases were removed automatically by EndnoteX7, preliminary screening based on the title and abstract was carried out by two authors (Jie Qian & Ying Wu) independently. The full texts of the literature above were downloaded and reviewed, followed by screening on the basis of the pre-established inclusion and exclusion criteria. For those studies for which consensus was not reached, even after discussion by both authors, a third member (Wan Yumei) decided whether to include the study or not.

### Inclusion and exclusion criteria

Inclusion criteria: (1) the epidemiological survey of the eating disorder evaluated a general population; (2) the diagnostic criteria of the eating disorder conformed to that of DSM or the ICD or the CCMD; (3) tools for screening were admitted; and (4) information such as prevalence and sample size in literatures could be extracted.

Exclusion criteria: (1) nonhuman studies, reviews, case reports and redundant published studies; and (2) studies on special populations, such as women, students, juveniles, hospitals and armies.

### Data extraction

For the included literature, data were extracted by Jie Qian and Ying Wu. The extracted items included first author; study year; study region; sampling method; diagnostic criteria; overall sample size; the number of cases; lifetime, 12-month, 3-month and 4-week prevalence rates; and sex.

### Evaluation of study quality

Similar to a previous study, the evaluation of the included literature followed the standards of the necessary items listed in Strengthening the Reporting of Observational Studies in Epidemiology (STROBE). Every item counted as a score of 1, with the total score ranging from 0 to 22.

### Statistical analysis

The Metaprop package in R version 3.6.2 software was applied, and Logit was used to perform rate conversion for consolidation computation. A heterogeneity test was performed. If *I*^2^ (statistics of effect value variation caused by heterogeneity) was less than 50% and the heterogeneity test *p* > 0.10, the fixed-effects model was adopted for rate consolidation; otherwise, the random-effects model was adopted. When there was significant heterogeneity among studies, sensitivity analysis was conducted. The funnel chart method was used to evaluate the publication bias of the included studies.

## Results

**Fig. 1:**
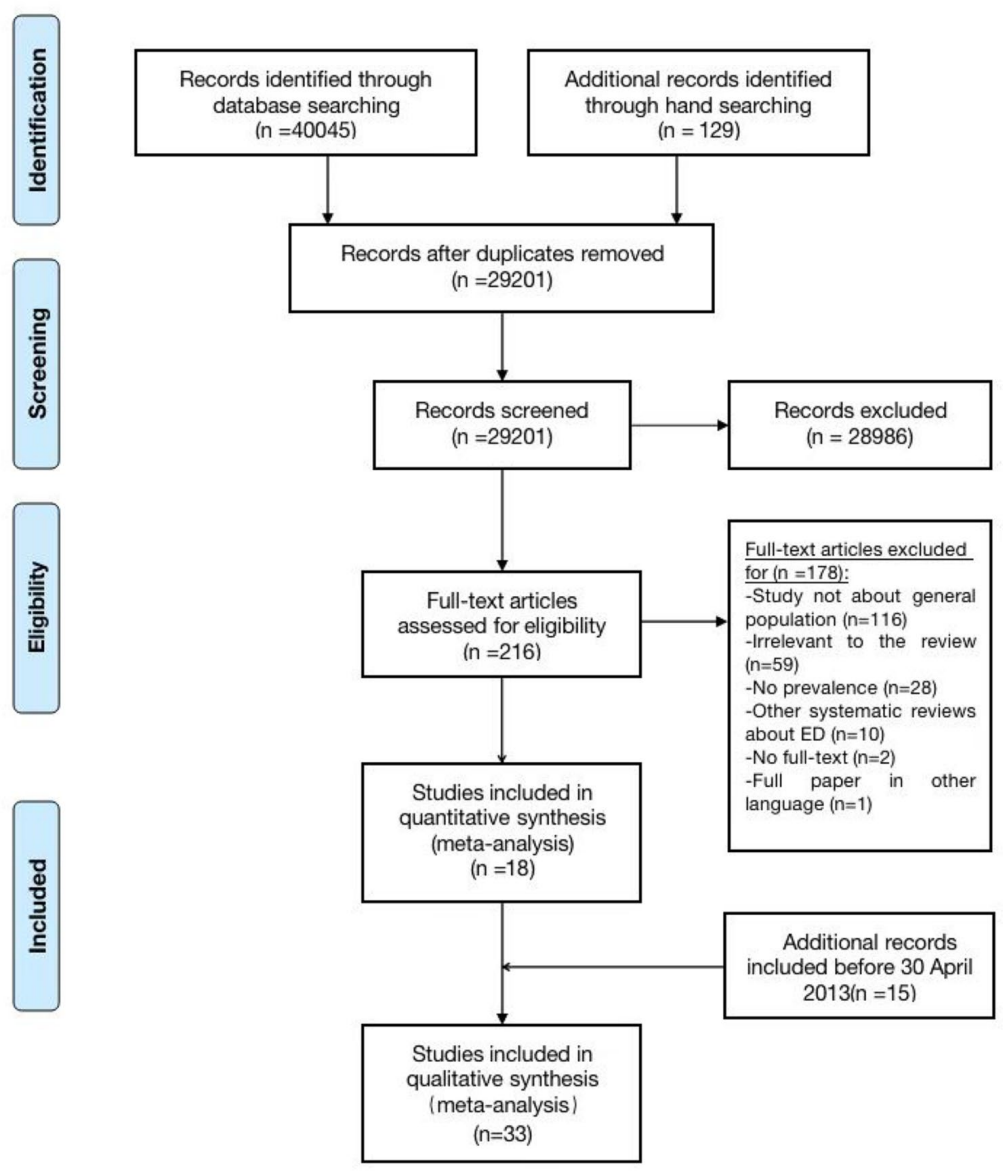
Identification of studies included in the analysis

A total of 29,201 unduplicated articles were retrieved at this time, 18,713 of which were in Chinese. After secondary screening, 18 studies were included according to the inclusion and exclusion criteria (Fig. 1).

### Characteristics of the included studies

**Fig. 2:**
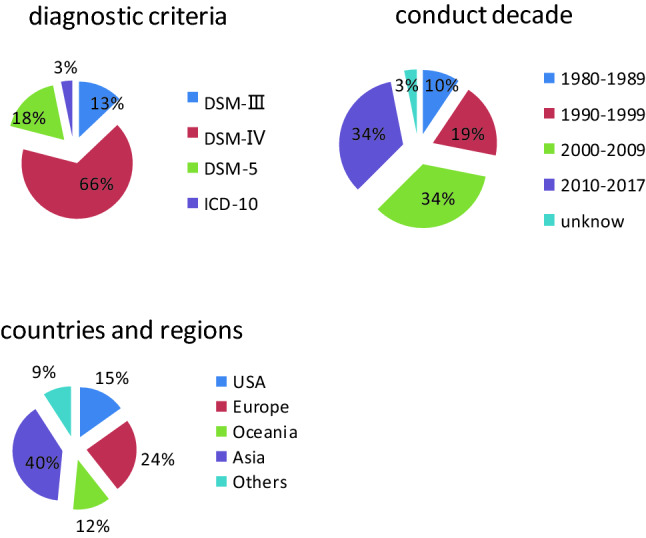
The proportion of different diagnostic criteria, conduct decade, countries and regions

Thirty-three studies were identified, which included 15 studies reported in the systematic review in 2013 [14]. Among the 18 included studies in this retrieval, 3 studies offered 2 or 3 groups of data: Hay 2017’s study [1] was performed in 2014 and 2015, respectively; Choi 2015’s study [21] was divided into KECR-R (performed in 2006–2007) and KECA-2011 (performed in 2011); and Shi 2015’s study [12] showed three groups of data from Shanxi Province, Gansu Province and Henan Province in China. Hence, there were 22 groups of data in total. The publication times of all studies ranged from 2002 to 2021, and the study times ranged from 2001 to 2017; however, the study time of one article published in 2014 was not specified [22]. There were 6 Chinese articles (including 3 dissertations [12, 23, 24]) and 13 separate English articles. Three studies were conducted in America [13, 18, 25]; 3 in Australia [1, 26]; 1 each in Italy [22], Switzerland [17], Korea [21] and Saudi Arabia [15, 16]; and 8 in China [12, 23, 24, 27–31], in which only 1 was national research [31], while the remaining 7 were regional studies limited to provinces or cities. DSM-IV and DSM-5 were used as newer diagnostic criteria than DSM-III and ICD. DSM-5 alone was applied in 4 studies, DSM-IV alone was applied in 11 studies, and DSM-IV and DSM-5 were both applied in the remaining 3 studies [22]. The cumulative total sample size of all studies was 242,917, with no subjects under the age of 15 years. There were only three studies involving EDs, of which one encompassed all types of eating disorders [13]; one included AN, BN and BED [17]; and the other did not specify the details of the eating disorders [12]. There were 15 studies referring to AN, 13 to BN and 10 to BED; other than that, there were two involving UFED, a new type in DSM-5, and one involving OSFED (Fig. 2).

The 33 studies included 315,877 participants. The studies were conducted from 1984 to 2017, with diagnostic criteria including DSM-III, DSM-IV, DSM-5 and ICD. The studies were conducted in the United States; European countries, including Germany, the Netherlands, Norway, Italy, Switzerland, Belgium, France, and Spain; Asian countries, including South Korea, China and Saudi Arabia; Oceanic countries, including Australia and New Zealand; and Latin countries, including Mexico [32] and Brazil [33]. In addition, there was an included study organized by the WHO involving 14 countries [34]. The specific percentages of study year, region and diagnostic criteria are shown in Table 2.

**Table 2:** Characteristics of the 18 Supplementary included studies

Study	Region	Sampling method^a^	Study time	Diagnostic criteria^b^	Sample size	Diagnosis^c^	Lifetime	Events^d^		
								12-m	3-m	4-w
Shi 2005	China (Zhejiang)	M,S,C	2001	DSM-IV	14,639	AN	–	–	–	2
Li 2008	China (Hebei)	R, M,S,C	2004–2005	DSM-IV	20,716	AN	3	–	–	3
						BN	1			1
Yu 2010	China (Guangzhou)	R,S,C	2009–2010	DSM-IV	2707	AN	1	1	–	1
Fang 2011	China (Fujian)	M,S,C	2009	DSM-IV	9986	AN	1	–	–	–
						BN	0			
						BED	0			
Liu 2012	China (Zhengzhou)	M,S,C	2011	DSM-IV	2 066	ED	37	14	–	–
Carta 2014	Italian	R	–	DSM-IV	3398	AN	24	–	–	–
						BN	20			
						BED	16			
Hay 2015	Australia	R	2008- 2009	DSM-5	6041	AN	–	–	28	–
						BN			40	
						BED			337	
						UFED			85	
Chio 2015	South Korea	M,S,C	Keca-R	DSM-IV	6510	AN	0*	–	–	–
			2006–2007			BN	7*			
			Keca 2011		6027	AN	0*			
			2011			BN	6*			
Shi 2015	China(Shanxi)	M,S	2013–2014	DSM-IV	901	ED	1	0	–	0
	(Gansu)				1014	ED	4	2		1
	(Henan)				1693	ED	4	4		3
Mohler-Kuo 2016	Switzerland	M,R,S	2010	DSM-IV	10,038	AN	72	5	–	–
				DSM-IV		BN	167	51		
				DSM-IV		BED	162	59		
				DSM-IV		ED	361	113		
				DSM-5		AN	109	5		
Cossrow 2016	USA		2013	DSM-IV	22,397	BED	340*	258*	–	–
				DSM-5		BED	455*	344	267*	
Duncan 2017	USA	S	2001–2003	DSM-IV	12,337	ED	466	225		–
						AN	31	2		–
						BN	186	78		–
						BED	278	145		
Wang 2017	China (Liaoning)	M,S,C	2014–2015	DSM-IV	19,733	AN	–	–		2
						BN				1
						BED				0
Hay 2017	Australia	S	2014	DSM-5	2732	AN	–	–	0	–
						BN			30	
						OSFED			0	
						UFED			0	
			2015		3005	AN			0	
						BN			37	
						OSFED			96	
						UFED			311	
Udo 2018	USA	M	2012–2013	DSM-5	36,309	AN	276	13	–	–
						BN	92	14		
						BED	318	166		
Huang 2019	China	M,C	2013–2015	DSM-IV	28,140	AN	8	1	–	–
						BN	5	4		
Altwaijri 2020	Saudi	M,S,C	2011–2016	DSM-IV	1981	AN	–	0	–	–
						BN	57*	42*		
						BED	63*	20*		
Bagaric 2020	Australia	S,R	2017	DSM-5	2977	BN	104*	–	33*	–
						BED	–		6*	

*The article did not provide the specific number of patients, but the prevalence, which was calculated according to

^a^*R* random, *C* cluster, *S* stratified, *M* multiphase

^b^*DSM* diagnostic and statistical manual, *ICD* International Classification of Diseases

^c^*ED* eating disorders, *AN* anorexia nervosa, *BN* bulimia nervosa, *BED* binge eating disorder

^D^*lifetime* lifetime prevalence, *12-m* 12-month prevalence, *4-w* 4-week prevalence

### Evaluation of study quality and the assessment of publication bias

The mean (sd) quality score for the reports of the 19 studies based on the STROBE items was 17.97 (1.71), and the range in this score varied from 15.5 to 22.0. Ten studies scored over 18.0, accounting for 52.6%, indicating high general quality of the literature. The most common problems in the reports of these studies were inadequate descriptions of the numbers of individuals in every phase, the reason for nonparticipation, and the numbers of individuals with missing data.

The assessment of publication bias on the prevalence of eating disorders and the three main types of EDs was conducted using the funnel chart method. The publication bias levels of eating disorders (*t* = − 1.97, *p* = 0.077), AN (*t* = − 0.21, *p* = 0.839), BN (*t* = 1.12, *p* = 0.278) and BED (*t* = 0.1, *p* = 0.921) were not statistically significant.

### Prevalence rates of eating disorders and the various types

**Fig. 3:**
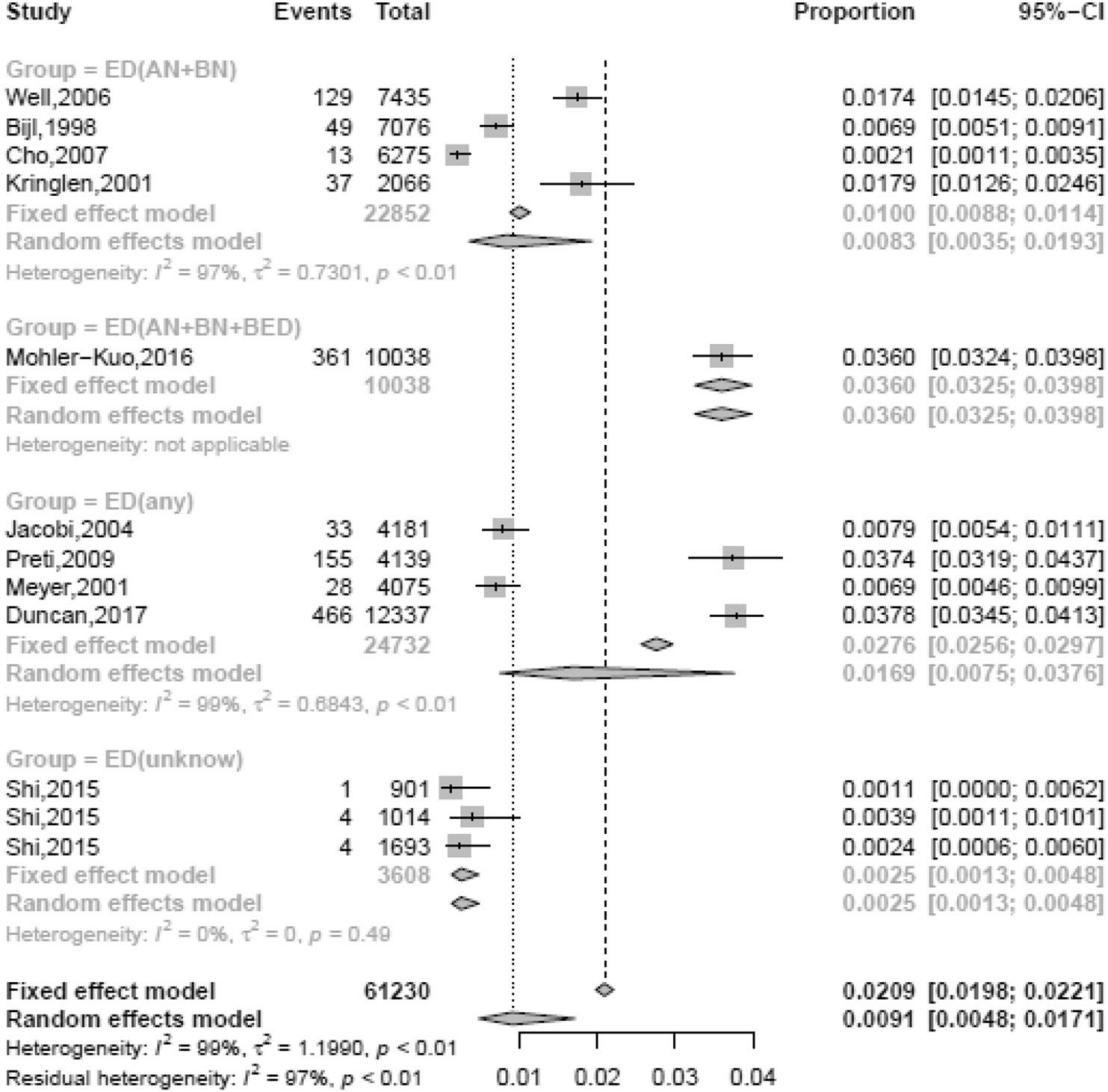
The lifetime prevalence of eating disorders

The lifetime, 12-month and 4-week prevalence rates of eating disorders were 0.91% (95% CI, 0.48–1.71), 0.43% (95% CI, 0.18–0.78), and 0.2% (95% CI, 0.09–0.36), respectively. As described above, the specific types of eating disorders in the 11 studies involving eating disorders were not completely the same. Hence, the 11 studies were categorized into 4 groups: ED (any), ED (AN + BN + BED), ED (AN + BN), and ED (unknown), representing the 4 studies including all types of eating disorders [13, 35–37]; 1 study including AN, BN and BED [17]; 4 studies [38–41] including only AN and BN; and 2 others without specific content [12, 42]. A lifetime prevalence of 3.60% and a 12-month prevalence of 1.1% were reported in the only study from the ED (AN + BN + BED) group. The lifetime prevalence rates of the remaining groups were 1.69% (95% CI, 0.75–3.76) for ED (any), 0.83% (95% CI, 0.35–1.93) for ED (AN + BN), and 0.25% (95% CI, 0.13–0.48) for ED (unknown). The lifetime and 12-month prevalence rates of ED (any) were over twice those of ED (AN + BN), and over 1.5 times those of eating disorders (shown in Table 3 and Fig. 3).

**Table 3:** Overall and subgroup prevalence of EATING DISORDERS

	Number of studies (number of datasets)	*n*	Number of cases	*I*^2^ (*p*-value)	Prevalence (%)	95% CI	*p*-value
Lifetime prevalence	10 (12)	61,230	1280	98.7 (< 0.001)	(R) 0.91	0.48–1.71	–
ED(any)	4 (4)	24,732	682	98.9 (< 0.001)	(R) 1.69	0.75–3.76	< 0.001
ED(AN + BN)	4 (4)	22,852	228	97.5 (< 0.001)	(R) 0.83	0.35–1.93	
ED(unknown)	1 (3)	3608	9	0.0 (0.435)	(F) 0.25	0.13–0.48	
DSM-IV	8 (10)	52,088	1194	98.8 (< 0.001)	(R) 1.17	0.46–2.20	–
DSM-5	0	–	–	–	–	–	
Studies conducted 1990–1999	4 (4)	17,398	147	87.5 (< 0.001)	(R) 0.91	0.59–1.31	0.005
Studies conducted 2000–2009	4 (4)	30,186	763	99.3 (< 0.001)	(R) 2.00	0.54–4.37	
Studies conducted 2010–2017	2 (4)	13,646	370	98.6 (< 0.001)	(R) 0.71	0.00–3.15	
Studies in Western countries	8 (8)	51,347	1258	98.5 (< 0.001)	(R) 1.89	1.03–3.01	< 0.001
Studies in Asia	2 (4)	9883	22	0 (0.623)	(F) 0.22	0.14–0.32	
Males	7 (7)	21,626	241	96.7 (< 0.001)	(R) 0.74	0.24 -1.52	0.034
Females	7 (7)	24,380	869	98.8 (< 0.001)	(R) 2.58	1.06–4.74	
12-month prevalence	10 (12)	61,230	489	96.9 (< 0.001)	(R) 0.43	0.18–0.78	–
ED(any)	4 (4)	24,732	290	98.0 (< 0.001)	0.72	0.15–1.71	< 0.001
ED(AN + BN)	4 (4)	22,852	80	92.8 (< 0.001)	0.34	0.11–0.69	
ED(unknown)	1 (3)	3608	6	66.8 (0.049)	0.10	0.00–0.38	
DSM-IV	8 (10)	52,088	448	97.4 (< 0.001)	(R) 0.41	0.13–0.83	–
DSM-5	0	–	–	–	–	–	
Studies conducted 1990–1999	4 (4)	17,398	62	69.3 (0.021)	(R) 0.36	0.59–1.31	0.776
Studies conducted 2000–2009	4 (4)	30,186	308	98.7 (< 0.001)	(R) 0.68	0.54–4.37	
Studies conducted 2010–2017	2 (4)	13,646	119	95.0 (< 0.001)	(R) 0.26	0.00–3.15	
Studies in Western countries	8 (8)	51,347	480	96.6 (< 0.001)	(R) 0.68	0. 34–1.13	< 0.001
Studies in Asia	2 (4)	9883	9	61.0 (0.053)	(F) 0.08	0.01–0.23	
Males	7 (7)	21,626	103	95.0 (< 0.001)	(R) 0.22	0.03–0.59	0.045
Females	7 (7)	24,380	330	96.7 (< 0.001)	(R) 0.93	0.37–1.74	
4-week prevalence	6 (8)	27,072	61	81.4 (< 0.001)	(R) 0.20	0.09–0.36	—

The lifetime prevalence rates of AN, BN and BED were 0.16% (95% CI, 0.06–0.31), 0.63% (95% CI, 0.33–1.02) and 1.53% (95% CI, 1.00–2.17), respectively. The lifetime prevalence of BN was nearly 4 times that of AN, and that of BED was nearly 10 times that of AN, with a ratio of approximately 1:4:10. The 12-month prevalence rates were also with great disparity, with a ratio of approximately 1:16:46. BED prevalence was markedly higher than the prevalence of the other two classic types, whereas AN prevalence was the lowest (Tables 4, 5 and 6).

**Table 4:** Overall and subgroup prevalence of ANOREXIA NERVOSA

	Number of studies (number of datasets)	*n*	Number of cases	*I*^2^ (*p*-value)	Prevalence (%)	95% CI	*p*-value
Lifetime prevalence	19 (21)	214,386	638	98.1 (< 0.001)	(R) 0.16	0.06–0.31	–
DSM-IV	14 (14)	148,372	245	97.0 (< 0.001)	(R )0.10	0.04–0.25	< 0.001
DSM-5	1 (2)	46,374	385	89.1 (0.002)	(R) 0.89	0.70–1.14	
Studies conducted 1980–1989	2 (2)	5100	1	35.2 (0.214)	(F) 0.01	0.00–0.06	0.200
Studies conducted 1990–1999	3 (3)	12,615	11	60.8 (0.078)	(R) 0.06	0.03–0.30	
Studies conducted 2000–2009	9 (10)	79,112	132	96.3 (< 0.001)	(R) 0.13	0.03–0.30	
Studies conducted 2010–2017	5 (6)	117,559	494	99.3 (< 0.001)	(R) 0.42	0.09–0.98	
Studies in Western countries	10 (11)	99,289	609	95.2 (< 0.001)	(R) 0.42	0.25–0.65	0.566
Studies in Asia	9 (10)	115,097	29	70.6 (< 0.001)	(R) 0.02	0.01–0.04	
Males	9 (10)	46,953	48	79.9 (< 0.001)	(R) 0.04	0.01–0.10	0.001
Females	9 (10)	49,866	508	96.6 (< 0.001)	(R) 0.62	0.29–1.08	
12-month prevalence	13 (14)	133,013	38	68.9 (< 0.001)	(R) 0.02	0.01–0.04	–
DSM-IV	10 (10)	80,107	20	58.2 (0.001)	(R) 0.02	0.01–0.05	0.187
DSM-5	2 (2)	46,347	18	0.0 (0.547)	(F) 0.04	0.02–0.06	
Studies conducted 1990–1999	3 (3)	12,615	0	–	–	–	0.591
Studies conducted 2000–2009	6 (6)	35,873	14	52.4 (0.062)	(R) 0.03	0.01–0.06	
Studies conducted 2010–2017	4 (5)	86,506	24	77.2 (0.002)	(R) 0.02	0.01–0.05	
Studies in Western countries	9 (10)	95,891	33	67.0 (0.001)	(R) 0.02	0.01–0.04	0.309
Studies in Asia	4 (4)	39,103	5	56.9 (0.073)	(R) 0.01	0.00–0.05	
Males	8 (9)	45,065	6	0 (0.493)	(F) 0.01	0.00–0.02	0.029
Females	8 (9)	47,203	23	70.1 (0.001)	(R) 0.03	0.00–0.06	
4-week prevalence	6 (6)	91, 506	10	0.0 (0.633)	(F) 0.01	0.00–0.02	–

**Table 5:** Overall and subgroup prevalence of BULIMIA NERVOSA

	Number of studies (number of datasets)	*n*	Number of cases	*I*^2^ (*p*-value)	Prevalence (%)	95% CI	*p*-value
Lifetime prevalence	19 (20)	198,102	1151	98.9 (< 0.001)	(R) 0.63	0.33–1.02	–
DSM-IV	13 (13)	135,624	855	99.2 (< 0.001)	(R)0.57	0.21–1.12	0.221
DSM-5	2 (2)	39,286	196	99.5 (< 0.001)	(R)1.41	0.00–6.30	
Studies conducted 1990–1999	3 (3)	12,615	66	60.8 (< 0.001)	(R) 0.53	0.06–1.49	0.476
Studies conducted 2000–2009	9 (10)	100,529	617	99.0 (< 0.001)	(R) 0.41	0.10–0.93	
Studies conducted 2010–2017	6 (6)	82,843	445	99.3 (< 0.001)	(R) 1.08	0.33–2.25	
Studies in Western countries	13 (13)	110,715	990	97.9 (< 0.001)	(R) 1.02	0.63–1.49	< 0.001
Studies in Asia	6 (7)	79,635	79	97.3 (< 0.001)	(R) 0.14	0.02–0.36	
Males	12 (12)	46,760	178	95.7 (< 0.001)	(R) 0.38	0.15 -0.73	0.008
Females	12 (12)	49,917	596	97.0 (< 0.001)	(R) 1.22	0.69–1.88	
12-month prevalence	13 (13)	146,373	408	97.4 (< 0.001)	(R) 0.31	0.15–0.53	–
DSM-IV	11 (11)	117,896	380	97.7 (< 0.001)	(R)0.29	0.12–0.54	–
DSM-5	1 (1)	36,309	44	–	0.14	–	
Studies conducted 1990–1999	3 (3)	12,615	42	96.6 (< 0.001)	(R) 0.29	0.00–1.11	0.942
Studies conducted 2000–2009	6 (6)	57,290	225	95 (< 0.001)	(R) 0.28	0.10–0.54	
Studies conducted 2010–2017	4 (4)	76,468	141	98.6 (< 0.001)	(R) 0.41	0.10–0.94	
studies in Western countries	10 (10)	102,225	324	94.5 (< 0.001)	(R) 0.33	0.19–0.51	0.294
Studies in Asia	3 (3)	36,396	46	98.6 (< 0.001)	(R) 0.27	0.00–1.20	
Males	8 (8)	40,488	52	90.0 (< 0.001)	(R) 0.09	0.02–0.22	0.113
Females	8 (8)	42,741	168	94.5 (< 0.001)	(R) 0.29	0.10–0.57	
4-week prevalence	5 (5)	53,064	30	94.8 (< 0.001)	(R) 0.07	0.00–0.23	—

**Table 6:** Overall and subgroup prevalence of BING EATING DISORDER

	Number of studies (number of datasets)	*n*	Number of cases	*I*^2^ (*p*-value)	Prevalence (%)	95% CI	*p*-value
BING EATING DISORDER							
Lifetime prevalence	11 (12)	151,320	2284	98.8 (< 0.001)	(R) 1.53	1.00–2.17	–
DSM-IV	9 (9)	89,216	1495	98.9 (< 0.001)	(R) 1.72	0.97–2.67	0.725
DSM-5	3 (3)	62,104	789	98.7 (< 0.001)	(R) 1.04	0.39–2.01	
Studies conducted 2000–2009	6 (6)	54,800	930	99.3 (< 0.001)	(R) 1.58	0.52–3.19	0.611
Studies conducted 2010–2017	5 (6)	96,520	1354	97.6 (< 0.001)	(R) 1.48	1.00–2.06	
Studies in Western countries	9 (10)	131,601	2048	96.8 (< 0.001)	(R) 1.57	0.82–2.55	0.358
Studies in Asia	2 (2)	11,967	63	99.5 (< 0.001)	(R) 0.80	0.00–6.86	
Males	9 (10)	54,720	481	96.1 (< 0.001)	(R) 1.17	0.73–1.73	0.037
Females	9 (10)	62,490	1357	94.2 (< 0.001)	(R) 2.42	1.91–2.99	
12-month prevalence	8 (9)	136,702	1250	96.5 (< 0.001)	(R) 0.93	0.66–1.24	–
DSM-IV	7 (7)	77,996	740	86.6 (< 0.001)	(R) 0.93	0.74–1.14	0.863
DSM-5	2 (2)	58,706	510	99.4 (< 0.001)	(R) 0.92	0.16–2.27	
Studies conducted 2000–2009	4 (4)	43,580	403	83.2 (< 0.001)	(R) 0.95	0.71–1.22	0.690
Studies conducted 2010–2017	4 (5)	93,122	847	98.1 (< 0.001)	(R) 0.90	0.49–1.44	
Studies in Western countries	7 (8)	126,969	1177	96.9 (< 0.001)	(R) 0.93	0.64–1.26	–
Studies in Asia	1	1981	20	–	1.00	–	
Males	6 (7)	51,871	276	94.0 (< 0.001)	(R) 0.51	0.28–0.82	< 0.001
Females	6 (7)	58,726	761	94.9 (< 0.001)	(R) 0.93	0.89–1.73	

Three studies involved the 3-month prevalence of AN, BN and BED, all conducted in Australia by Hay et al., with DSM-5 as the diagnostic criteria used. The 3-month prevalence rates of AN were 0.46% during 2008–2009 and 0 in both 2014 and 2015. The 3-month prevalence of BN was 0.99% based on pooled data of 4 groups from the 3 studies. The 3-month prevalence of BED was 1.71% based on pooled data from 3 studies.

The 3-month prevalence of OSFED and UFED was seen only in the report conducted by Hay et al. The 3-month prevalence rates of OSFED were 0 in 2014 and 3.2% in 2015. The 3-month prevalence of UFED was 2.2% based on the pooled data from the 2 studies.

Sensitivity analysis of the main results was conducted, and no study with a significant change in heterogeneity was found.

### Subgroup analysis

#### Diagnostic criteria

Subgroup analysis using the diagnostic criteria of DSM-5 and DSM-IV was added in this report. No study involving EDs used DSM-5 diagnostic criteria. The pooled lifetime prevalence of AN using DSM-5 was 0.89%, which was 8.9 times that using DSM-IV (*p* < 0.001); the pooled 12-month prevalence was 2 times that using DSM-5 than that using DSM-IV. For BN, the pooled lifetime prevalence using DSM-5 was 1.41%, which was 2.5 times that using DSM-IV. However, for BED, the lifetime and 12-month prevalence rates diagnosed by DSM-5 were slightly lower than those diagnosed by DSM-IV, but the differences were not statistically significant.

#### Years of study

**Fig. 4:**
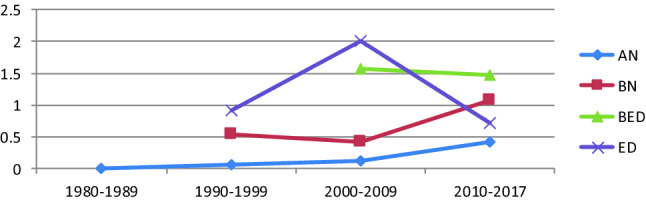
The lifetime prevalence of eating disorders over time

The lifetime prevalence rates of EDs in the 1990s, 2000s and 2010s were 0.91%, 2.0% and 0.71%, respectively, while the 12-month prevalence rates were 0.36%, 0.68% and 0.26%, respectively. The highest prevalence was in the 2000s for both conditions, with a lower prevalence in the 2010s. The lifetime prevalence rates of AN in the 1980s, 1990s, 2000s and 2010s were 0.01%, 0.06%, 0.13% and 0.42%, respectively, with an upward trend. The lifetime prevalence rates of BN in the 1990s, 2000s and 2010s were 0.53%, 0.41% and 1.08%, respectively, and the 12-month prevalence rates were 0.29%, 0.28% and 0.41%, respectively. The lifetime prevalence rates of BED in the 2000s and the 2010s were 1.58% and 1.48%, respectively, and the 12-month prevalence rates were 0.95% and 0.90%; the prevalence rates in the 2010s were both lower than those in the 2000s for both conditions (shown in Fig. 4).

#### Geographic region

**Fig. 5:**
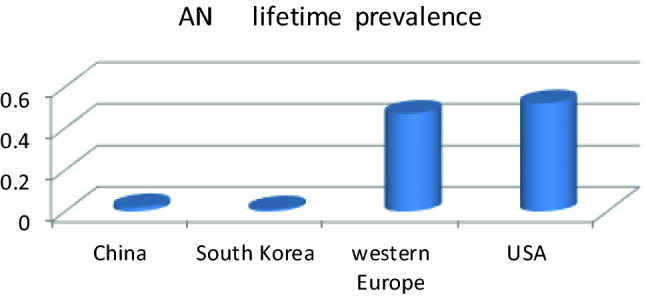
The lifetime prevalence of AN in different countries or regions

We used the same method for comparing Western and Asian countries as in a previous report. The lifetime and 12-month prevalence rates of EDs in Western countries were 1.89% and 0.68%, respectively, both over 8.5 times those in Asian countries (*p* < 0.01). The lifetime prevalence of AN in Western countries was 21 times that in Asian countries, 7.3-fold for BN and 2-fold for BED. The 12-month prevalence rates were 2-fold and 1.2-fold higher in Western countries than in Asian countries for AN and BN, respectively. The prevalence in Western countries was significantly higher than that in Asia for any type of eating disorder. Furthermore, the pooled lifetime prevalence rates of AN were subdivided by region of South Korea, China, Western Europe and America, with results of 0.01%, 0.02%, 0.47% and 0.52%, respectively (shown in Fig. 5).

#### Sex

The lifetime prevalence rates of eating disorders in females reached 2.58% (95% CI, 1.06–4.74) and 0.74% (95% CI, 0.24–1.52) in males. Regardless of overall eating disorders or various types, the prevalence in females was evidently several times that in males. The lifetime eating disorder prevalence rates at 12 months and 4 weeks in females were 3.5, 4.2 and 3.1 times those in males, respectively. The ratios of females to males in the lifetime prevalence of AN, BN and BED were 15.5, 3.2 and 2.1, respectively, and 3, 3.2 and 1.8 in the 12-month prevalence. Compared with the classic types AN and BN, the ratio was lower for BED.

## Discussion

### Main finding

This study updated the prevalence of eating disorders in the general population before 2021, and analyzed the different conditions included among eating disorders to obtain closer-to-real-level prevalence data. The differences in sex, region, decades and diagnostic criteria were analyzed.

After this update, the pooled lifetime and 12-month prevalence rates of eating disorders were 0.9% and 0.43%, respectively. However, the pooled lifetime and 12-month prevalence rates of ED (any), the subgroup of which covers all types of eating disorders, were 1.69% and 0.72%, respectively, both over 1.5 times those of the eating disorders among all 12 groups of data. Many studies on the prevalence of eating disorders included only the 2 classic types, AN and BN, whereas their prevalence rates were comparatively lower among various types. This reflected the situation in which the prevalence of eating disorders in the general population was underestimated. Another factor that needs to be considered is that the 4 studies of EDs (any) all come from Western high-income countries (Germany, the United States and six other European countries).

The lifetime prevalence rates of AN, BN and BED were 0.16%, 0.63% and 1.53%, respectively, which were all lower than the results in a previous report from 2013. This may be related to the fact that only three studies were from South Korea, the only Asian country included in the previous report, whereas a large number of Asian country studies, especially China, were included in this report.

To date, few studies have used DSM-5 to study the prevalence of eating disorders. Thus, studies that use DSM-5 diagnostic criteria to conduct comprehensive investigations of all types of eating disorders are still lacking. The prevalence of AN using the diagnostic criteria of DSM-5 was much higher than that using DSM-IV, in accord with other study results [19, 20, 43]. This was also in line with the fact that the standards of AN experienced the greatest alteration in DSM-5 diagnostic criteria. For BN, the lifetime prevalence using DSM-5 was higher than that using DSM-IV, in accord with others studied by Lindvall Dahlgren [43] and Flament [19], which could be because the frequency of the diagnosis was reduced. However, Mancuso [20] showed that the prevalence of BN was not different using the two diagnostic criteria and used only self-report questionnaires for diagnosis in adolescents. For BED, although the standards of the frequency of binge eating were relaxed in DSM-5, there was no obvious evidence that the prevalence using DSM-5 was higher than that using DSM-IV. Thus, the conclusions in other studies were in discordance. Lindvall Dahlgren [43] reported that there had been early evidence indicating a higher prevalence of BED using DSM-5. Flament [19] found that the prevalence of BED had no change in juveniles. More research using DSM-5 is needed for comparison in the future.

Except for AN, the prevalence of other types of EDs did not significantly increase with time until the 2010s. The prevalence of EDs in the 2000s showed an increase compared with that in the 1990s, which was in accordance with Mitchison's [44] and Hay's [11] studies on the prevalence trends of eating disorders. However, the prevalence declined instead of growing in the 2010s. Nevertheless, more studies need to be included, as only 2 studies were included after 2010 [12, 17]. The upward trend in AN and the slight downward trend in BN were in line with most other studies [4, 45, 46], but studies of new types of eating disorders are still lacking.

In terms of the distributions of geographic region and sex, the results in this report were consistent with those of a previous study by the authors and others, in which the prevalence rates in Western countries and in females were higher. The lifetime prevalence rates were up to 2.58% in females and 1.89% in Western countries, higher than the prevalence rates of schizophrenia and other mental illnesses. The real prevalence rates in Western countries and in females might be even higher due to underestimation, as mentioned above. Although the prevalence was higher in females, male patients showed a slightly higher proportion of BED than of the classic types AN and BN, consistent with the results of Smink's study [4].

### Strengths and limitations

There have been few reviews about the prevalence rates of eating disorders in the general population. Of the 15 studies included in our former meta-analysis, only 3 studies of one country (South Korea) were from Asia. However, 8 Chinese studies, accounting for 50% of all newly included studies, were included in this report. As the specific contents of eating disorders varied greatly in different studies, subgroup analysis was conducted based on the specific contents, which might provide more authentic data. The number of studies was more than double, and most of the main results were calculated based on more than 10 studies, with publication bias assessed by the funnel chart method, which essentially resolved the limitations of previous reports.

However, the number of studies on the prevalence of eating disorders is still small, concentrated in Europe, America, Australia, New Zealand and a few Asian countries, such as China and South Korea. Studies from other countries were not retrieved, probably due to language limitations, and the distribution of geographic regions cannot be analyzed in a larger scope. The number of studies using DSM-5 was small, with no study covering all types of eating disorders found in DMS-5. Likewise, those of AN, BN and other types were also very small; thus, it is impossible to conduct a comprehensive analysis on the impact of the new diagnostic criteria on the prevalence of eating disorders.

### Conclusion

The prevalence of eating disorders may be underestimated thus far. A large number of previous epidemic studies did not include all types of eating disorders, whereas the prevalence in new types was apparently higher. The prevalence of eating disorders was high, especially in Western countries and in females. In addition, we should pay more attention to new types of eating disorders in males. In future epidemic investigations, new diagnostic criteria should be used more often to comprehensively evaluate all types of eating disorders.

## Supplementary information

### What is already known on this subject?

Only one systematic review on the prevalence of EDs in general population which was done by us in 2013. There have been some epidemiological studies of EDs in recent years with mixed results.

### What this study adds?

The prevalence of eating disorders might be underestimated. Not all types of EDs were included in epidemiological surveys. New diagnostic criteria shall be used more to access EDs comprehensively.

## Data Availability

All data generated or analyzed during this study are included in this published article (and its supplementary information files).
